# Schools as Neighborhoods: A Holistic Framework for Student Well-Being, Opportunity, and Social Success

**DOI:** 10.3390/children13010059

**Published:** 2025-12-31

**Authors:** Cordelia R. Elaiho, Constance Gundacker, Thomas H. Chelius, Brandon Currie, John R. Meurer

**Affiliations:** 1 School of Medicine, Medical College of Wisconsin, Milwaukee, WI 53226, USA; 2 Department of Pediatrics, Medical College of Wisconsin, Milwaukee, WI 53226, USA; 3 Institute for Health & Humanity, Medical College of Wisconsin, Milwaukee, WI 53226, USA; 4 STRYV365, Milwaukee, WI 53202, USA; bcurrie@stryv365.org

**Keywords:** school, adolescents, social capital, neighborhood, socioecological model, social-emotional learning, trauma-informed curricula, school-based health

## Abstract

Background: Schools play a central role in child development and socialization and can function as protective environments that mitigate the effects of adversity. Building on the Social Ecological Model and Community School Transformation, we propose a “Schools-as-Neighborhoods” framework that conceptualizes schools as intentionally designed microenvironments capable of generating social capital, promoting positive childhood experiences, and buffering harmful neighborhood exposures through trauma-informed programming. Methods: We conducted a convergent mixed-methods study across four public and charter schools in Milwaukee, Wisconsin, serving grades five through nine. STRYV365’s *peak team* and Brain Agents gamified intervention were implemented between 2022–2024. Quantitative surveys and qualitative data assessed students’ lived experiences, exposure to adversity, emotional awareness, coping skills, and school connectedness/climate across multiple waves. Results: Across the four schools (*n* = 1626 students), baseline academic proficiency was low, and exposure to adversity was high among surveyed participants (*n* = 321), including bereavement (74%) and family incarceration (56%). Despite these challenges, qualitative findings revealed strengthened emotional regulation, empathy, motivation, and goal setting among students engaged in trauma-informed programming. Teachers reported improved peer interaction and community building during sustained implementation. Conclusion: The Schools-as-Neighborhoods framework highlights the value of trauma-informed, relationship-centered school environments in promoting student well-being. By positioning schools as cohesive ecosystems that foster belonging and cultivate social capital, this approach offers educators and policymakers a pathway for mitigating the effects of hostile lived environments and supporting students’ mental health, social development, and engagement in learning.

## 1. Introduction

Education plays a critical role in shaping children’s health, development, and long-term opportunities. Yet persistent disparities in access and outcomes highlight the need to reimagine how schools support students, particularly those facing adversity. Various proposals for bettering neighborhood schools exist. Most involve bettering student and community relations; however, for students in underserved communities and under-resourced schools, the community does not always provide appropriate avenues for self-improvement. Schools provide a unique opportunity to function as a community and neighborhood in their own right, helping to counteract unproductive environments and enhance students’ physical and mental well-being.

### 1.1. Government Involvement in Education

States spend a considerable amount of funding on education, plus an additional amount in United States Federal contributions, with the Department of Education (DoE) having the third-largest discretionary fund [1]. However, this quality and administration of education has caused divisions in scholastic achievement, resources, financial mobility, and health. Urban and lower-resourced schools experience higher rates of truancy, dropout rates, lower-quality education, and lower health outcomes [2].

The DoE and public education at the state and local levels in the United States have long been a topic of contention [3]. The DoE was initially created to collect and condense information about American schools to inform school system effectiveness in teaching and create globally competitive students. Since its origins, the DoE has been responsible for managing federal student loans and ensuring that there is federal support for students in under-resourced areas [1]. Various innovations have occurred to assist these differences, including the Elementary and Secondary Education Act of 1965, the No Child Left Behind law signed in 2002, and the Every Student Succeeds Act signed in 2015 [1,3,4,5]. However, with each administration, opposing bureaucratic ideals and rhetoric cause inconsistency and slowed progression for students [6,7,8]. Education is a social driver of health metric, with high school diplomas and higher education allowing for social advancement, adaptability, and increased access, which improve health and economic outcomes [9]. Yet, there has been a continuous achievement gap in under-resourced schools, with shifting national priorities creating challenges for continuity in trauma-informed and social–emotional programming.

### 1.2. Frameworks for Expanding the Role and Redefining Schools

**Figure 1:**
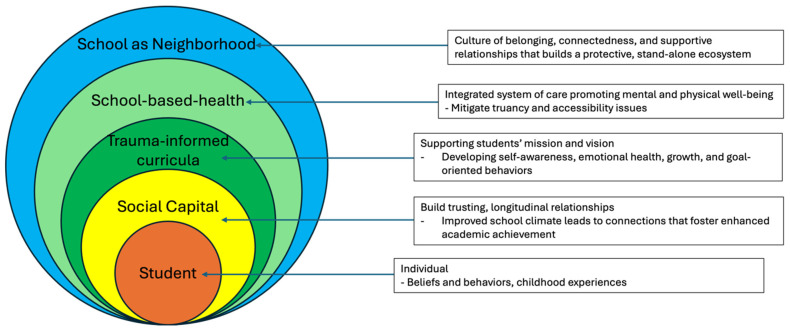
Schools-as-Neighborhoods conceptual framework and key concepts.

A sociological definition of a neighborhood incorporates the immediate geographical location of a residence, including physical features and social interactions, cohesion, and shared identity of the neighborhood’s individuals [10]. The impact of neighborhoods on the development, lifestyle, and prospects of children and adolescents has been documented in topics including, but not limited to, poverty, crime and violence, socialization, and education [11,12]. The Social Ecological Model (SEM) is a framework that identifies how interpersonal relationships, institutions and organizations, the community, and broader structures and systems impact an individual’s attitudes, beliefs, knowledge, behaviors, and overall health [13]. Resourced and socially advancing neighborhoods typically have well-funded institutions with like-minded individuals, which increases social cohesion and social capital. These things being considered, it is paramount to view and orchestrate schools as not just in neighborhoods, but as their own ecosystem/neighborhood within the neighborhood. This allows for a shift in mindset, resources, and outcomes for the school. Schools-as-Neighborhoods is a conceptual framework that defines schools as intentionally designed micro-environments that function as protective neighborhoods for children and adolescents, particularly those facing adversity. The framework emphasizes the school itself as a primary site for generating social capital. The framework distinguishes schools as a standalone community that supports the health, mission, and vision of its students through relationship-centered practices (Figure 1). This contributes to positive childhood experiences (PCEs). By reframing the notion of schools through this framework, schools are positioned as modifiable environments buffering harmful neighborhood exposures while supporting emotional, social, and developmental well-being. Few manuscripts describe the concrete notion of Schools-as-Neighborhoods and social shifters. There are elements of these aspects in social capital and in physical and mental health school resources, such as the Community School Transformation (CST) framework; however, this manuscript seeks to define and reframe schools as standalone neighborhoods that can positively impact students beyond written knowledge [14].

Utilizing data from students in Milwaukee, WI, USA, in grades five through nine, and following them into the next school year, we identify vulnerable youth and schools and examine whether trauma-informed interventions—*peak team* and Brain Agents—foster student emotional well-being and school connectedness. Additionally, through this work, we propose a framework of Schools-as-Neighborhoods, building on the community school model [15], in which schools function as cohesive ecosystems supporting social capital, emotional health, and community belonging. With proposed funding cuts to the DoE, it is essential that schools create sustainable and progressive frameworks that support students independent of curriculum, funding, or ideological changes [7,16,17].

## 2. Materials and Methods

### 2.1. Literature Review

This study incorporates a scoping review to better understand the individual concepts of a neighborhood and the elements in practice within adolescent education. A literature search utilizing PubMed, the Education Resources Information Center (ERIC), which is managed by the United States Department of Education, SCOPUS, and Google Scholar, assisted in identifying the breadth of literature. Search terms are located in Appendix A, Table A1.

Search results center around urban centers and schools, and the myriad of factors that impact the development of a child/adolescent and the makeup of an advancing neighborhood, including: social capital, health/school-based health, and educational policies. The Community Schools Transformation framework outlines key practices for community schools with a culture of belonging, integrated support, student and family engagement, and expanded learning opportunities.

### 2.2. Implementation of a Trauma-Informed Curriculum at Four Milwaukee Schools

#### 2.2.1. School Participants and Context

STRYV365 is a non-profit organization based in Milwaukee, Wisconsin, designed to customize non-clinical trauma-informed curricula. STRYV365 utilizes multifaceted approaches to curriculum design, fostering resilience and increasing positive lived experiences for school-aged children. Two SAMHSA-based social–emotional learning curricula, the *peak team* coaching program and the Brain Agents video game, were developed by STRYV365. Both curricula utilize the COPE (Creativity, Optimism, Planning, and Expert Information) and CRAFFT (Care, Relax, Alone, Forget, Family/Friends, and Trouble) frameworks and incorporate physical activities and reflective art-based exercises to promote student self-awareness, decision-making, and relationship building [18,19,20,21]. STRYV365 partnered with four schools in Milwaukee: two public and two charter.

#### 2.2.2. Study Design and Conceptual Framework

This study employed a convergent mixed-methods design in which quantitative survey data and qualitative focus group and interview data were collected in parallel across four semesters and integrated at the interpretation stage. Quantitative analyses tested prespecified hypotheses regarding social–emotional learning (SEL) outcomes using a clustered randomized incomplete block factorial design (Appendix B Table A2), with classrooms as the unit of randomization. Qualitative analyses explored students’ and teachers’ lived experiences of emotional regulation, coping, and school connectedness. Integration occurred through thematic triangulation, whereby quantitative findings for each SEL domain (e.g., emotional regulation, coping strategies) were presented alongside corroborating or explanatory qualitative themes.

Intervention fidelity was supported through standardized curricula, trained coaches, and consistent implementation frameworks across schools. *Peak team* student-coach sessions followed a structured trauma-informed curriculum aligned with the COPE and CRAFFT frameworks. It was delivered over 9–12 sessions within 4–10 weeks, with flexibility to accommodate school schedules and student needs. Brain Agents provides complementary trauma-informed support through the gamification of narrative contexts and minigames. It followed a standardized schedule of 2–3 sessions per week for 4–5 weeks, with usage metrics captured directly from the game platform. Variation in duration and exposure was addressed analytically through the inclusion of cluster indicators.

Sociocultural theory informs the conceptual framework for the study [22,23]. The mixed-methods design operationalizes the Schools-as-Neighborhoods concept by measuring emotional, behavioral, and relational outcomes through surveys and qualitative interviews. We hypothesized that two of STRYV365’s school-based programs, the *peak team* coaching program and complementary Brain Agents video game interventions, helped establish and reinforce a safe, supportive, neighborhood/community-building environment that modifies student feelings, attitudes, behavior, and school performance, originally influenced by their lived environment. Research questions included: What did success mean to each of the students, and how did students intend to obtain their goals? How do trauma-informed programs influence students’ emotional awareness, coping skills, and school connectedness? How do teachers perceive changes in classroom relationships after implementation?

This work was submitted to and approved by the Medical College of Wisconsin Institutional Review Board (PRO00037500) in Milwaukee, WI, USA.

#### 2.2.3. Recruitment and Participant Consent and Assent

The two interventions were implemented in four Milwaukee, Wisconsin schools for students in grades five through nine between 2022–2024. Students were randomized to one of four groups: Brain Agents, *peak team*, both, or neither intervention. STRYV365 and school staff were CITI-trained in human research protection. Parents and caregivers of students at each school were recruited at back-to-school events, open houses, parent-teacher conferences, and through emails. Informed consent was obtained on digital tablets in English for study participation. Students provided informed assent before completing the baseline REDCap survey and joining focus groups or interviews [24].

#### 2.2.4. Quantitative Analysis

Sample size considerations were informed by a priori power analysis conducted for the primary SEL outcomes. To detect small-to-moderate effects (Cohen’s d = 0.3) with 80% power and α = 0.05 across four intervention conditions, approximately 175 participants per arm were estimated to be required. Although consent and assent rates resulted in a smaller analytic sample, the repeated measures clustered design increased statistical efficiency. Analyses therefore emphasize effect estimation with appropriate covariate adjustment rather than reliance on dichotomous significance testing alone.

Data collection included a 41-question survey instrument in REDCap derived from validated and reliable items provided by the Life Paths Research Center and aligned with the competencies outlined by the Collaborative for Academic, Social, and Emotional Learning [25,26]. The surveys administered in the fall, winter, and spring of each intervention year assessed key SEL domains such as self-awareness, self-management, relationship skills, responsible decision-making, social awareness, and adversity. The surveys also included recent feelings of depression and anxiety, using items from the Patient Health Questionnaire-2 and Generalized Anxiety Disorder-7 questionnaires [27,28]. Students who reported daily symptoms of anxiety or depression were promptly referred to their school behavioral health counselor and a child psychologist through STRYV365.

Quantitative analysis used mixed-effects regression models with random intercepts for classroom and school to account for clustering and repeated measurements over time. Assessments were conducted at baseline (early fall 2022) and at subsequent waves in late fall, spring, and the following academic year (through spring 2024), corresponding to intervention cycles. Of the 1626 students enrolled across the four schools, 321 consented to complete surveys. Response rates varied by wave, with 225–277 respondents per wave, 70–86% of invited participants. Attrition across waves primarily reflected student absenteeism and school mobility rather than study withdrawal. Rather than imputing missing outcomes, analyses used all available data under a missing-at-random assumption, incorporating baseline scores, cluster indicators, and time effects to reduce bias. This approach is appropriate for longitudinal school-based studies with staggered participation and minimizes distortions that may arise from imputation in the presence of structural absenteeism.

#### 2.2.5. Qualitative Analysis

Qualitative data were collected through semi-structured student focus groups, individual student interviews, and teacher focus groups conducted at the conclusion of intervention semesters. Student focus groups for fifth through seventh grade lasted 45–60 min and were designed to promote peer interaction, while individual interviews with older students, 8th–10th grade, were 30–45 min and allowed for deeper reflection. Teacher focus groups were 60 min in length. Focus group prompts addressed emotional regulation, coping strategies, future aspirations, relationships, school connectedness, and perceptions of program impact (Appendix C). Qualitative data included focus groups with 57 students, individual interviews with 68 students, and focus groups with 29 teachers and school staff.

Qualitative analysis of focus group and interview transcripts used a hybrid deductive-inductive thematic coding approach. An initial deductive codebook was developed based on the same CASEL and emotional intelligence domains as the quantitative analysis. Inductive codes were then added iteratively as novel concepts emerged from the data. Each transcript was independently coded by two coders, with discrepancies resolved through consensus meetings, minimizing bias and enhancing analytic rigor. Thematic saturation was considered achieved when successive focus groups and interviews failed to generate new codes within the predefined SEL/EL domains, and when thematic patterns remained consistent across schools, grade levels, and intervention conditions.

## 3. Results

### 3.1. Quantitative Findings

#### 3.1.1. School Statistics and Participant Demographics

A total of 1626 students in grades five through nine collectively attend the four schools. Of those who participated in the interventions of *peak team* and Brain Agents, 61% were Black/African American, 19% White, 11% Hispanic/Latinx, 6% Asian/Hmong, and 3% mixed racial/ethnic background. 69% experience economic disadvantage. In state standardized exam performance, 58% were below basic, 27% basic, and 15% were proficient in English and language arts and math, respectively. 28% were chronically absent, and 88% of students at the schools earn high school diplomas. Baseline statistics of the four schools depict that the majority of students come from economically disadvantaged communities. Economically disadvantaged youth in Milwaukee are defined by direct certification or household income guidelines for free or reduced-price meals under the National School Lunch Program (NSLP) [29]. These schools do not have a school-based health center, integrated alternative, or trauma-informed curriculum. Additionally, the schools have, on average, a 25% chronic absenteeism rate; however, three of the schools have an average 90% graduation rate, while one school has a graduation rate of 47% at baseline (Appendix C Table A3). A subset of participants, *n* = 321, were invited to complete a survey indicating their exposure to adversity. Their demographics are depicted in Appendix C Table A4.

#### 3.1.2. Student Adversity and Resilience

Up to 277 students responded to questions about adverse events. Cyberbullying items were added in fall 2024 and not collected in earlier semesters. At baseline for the first group of participants, in early fall 2022, 74% of students had someone close to them die, 56% had someone close to them go to jail, and almost 30% had someone close to them with substance use issues. About one-third had felt nervous or anxious early every day for a two-week period. About one-fifth had signs of melancholy (Table 1). Notable trends included a decrease in the proportion of participants reporting symptoms of depression or anxiety following implementation of the interventions. Survey responses were analyzed as a cross-sectional series of different students over two years.

### 3.2. Qualitative Findings

#### 3.2.1. Emotional Awareness and Regulation

Quotes from the qualitative analysis of student focus group and interview data are recorded in Table 2. Participants were asked how the trauma-informed curriculum of *peak team* and Brain Agents helped their emotional awareness, coping mechanisms, future goals, and school connectedness. The trauma-informed learning emphasized student qualities and goals. Students identified intrinsic and extrinsic qualities such as resilience (Quote 4, Table 2), self-regulation (Quote 5, Table 2), and physical activity or spiritual discipline (Quote 6). Notably, participants’ future goals spanned education, financial stability, and creating a meaningful life (Quotes 10–12, Table 2). Some students noted that their neighborhood cohesion was lacking outside of school and identified a seeming barrier for social mobility (Quote 15, Table 2). However, students highlighted their anticipation and desire to engage with STRYV365 programming while at school, differing from their outside experience (Quotes 1–2, Table 2).

#### 3.2.2. School Connectedness and Climate

Teachers were asked to evaluate their understanding of the *peak team* and Brain Agents curricula, noticeable changes in the students, and suggestions for the rollout of the program. Overall, teachers expressed the connection that students make with the coaches of the STRYV365 program. The longitudinal engagement and recognizability of the coaches support the students and the school administrators through stressful times. Consistent coaching and engagement fostered belonging and a positive classroom culture. Teachers noted changes in student behavior when STRYV365 programming was in session. In Quote 18 of Table 3, a teacher expressed how the implementation of the program helped the culture of the school. The students were “high-fiving, greeting coaches, and excited” throughout the school day, but especially after a session. Another teacher reported early-year challenges and observed improvements following implementation of the *peak team* and Brain Agents programming, although she could not scientifically attribute these changes to the intervention (Quote 19, Table 3). One teacher noticed a temporal nature to difficulties with students, noting that “during holidays, breaks, or earlier in the year,” when students are away from the school and the intentional curriculum, “you get these more infractions… we’ve had a brawl at high school” (Quote 21, Table 3).

## 4. Discussion

### 4.1. STRYV365 Curriculum for Students in At-Risk Schools

This study explored how trauma-informed programs can enhance emotional well-being and connectedness within the conceptual framework of Schools-as-Neighborhoods. Our study of adolescents in four Milwaukee middle and high schools assessed risk profiles and social relationships of vulnerable youth. We utilized a trauma-informed care curriculum to assess their ability to manage their emotions and have better interpersonal outcomes. These skills are a necessary part of education and social mobility.

These four schools ranged in student population size from small to mid-sized, and from true public to charter schools. Another option for parents and students is private choice, which allows parents to use public funds to enroll their students in private schools. These schools generally have smaller class sizes and alternative or expanded curricula [30]. Previous studies suggest that smaller schools and class sizes promote better student outcomes, with the flexibility of charter or private choice schools providing a more supportive or personalized environment [30,31]. Despite these indicated improvements, the schools in this study still had the majority of students well below proficiency in English/Language Arts and Mathematics, and an over 20% chronic absenteeism rate (Appendix C Table A3). This would suggest that class size and piecemeal policies are insufficient to provide the successful student and ultimately, citizen, standardized education purports. Despite these statistics, three of our schools have an average 90% graduation rate. These statistics warrant deeper scrutiny of the curriculum and metrics for graduation. Although this study combines results from the four schools, focusing on their similarities, notable differences among the schools exist regarding racial demographics and academic achievement. These are most remarkable at schools with the greatest economic disadvantage (Appendix B). This requires discussion of racial and economic inequities beyond the scope of this study; however, it furthers the basis for expanded social capital and mitigating environments for PCEs, provided by the Schools-as-Neighborhoods framework, particularly for vulnerable youth.

The STRYV365 curriculum fosters longitudinal relationships, promotes goal-oriented behaviors, and establishes a structure for self-awareness and positive childhood experiences (PCEs). The *peak team* methodology consisted of trained coach-mentors holding weekly group sessions focused on reframing experiences and interactions, broadening beliefs and dreams, and providing tools for refocusing. Physical activities to foster team building and increase social capital included team sports such as basketball and soccer, multi-player games, reflective writing, and group discussions. The Brain Agents video game similarly fostered resilience in the tasks completed to achieve new game levels and reflections on feelings identified while playing. In Table 2, student participants expressed personal goals and individual characteristics they desired to develop and utilize for social mobility. This additional curriculum offered in schools to students with adverse risk factors supports students not only in their typical education but also in soft skills, which promote the development of the whole individual. Understanding conflict resolution, goal setting, and relationship building are skills that members of a neighborhood use to create safety and growth of the neighborhood and its individuals. Identifying schools as neighborhoods and reframing the current resources and metrics for adolescent success can assist with producing not only informed, but also socially competent and overall healthy graduates [32].

### 4.2. Schools as Neighborhoods

The impact of the lived environment on adolescents is well documented [9]. Schools, according to the SEM, would be considered one variable in the social context that affects a person. However, since, according to the National Center for Education Statistics, children spend an average of seven hours per day over 180 days in school, these communities can be considered their own neighborhoods [33,34]. Thus, our proposed framework (Figure 1) illustrates that belonging, connection, and supportive relationships function as protective layers within the school environment, reinforcing students’ resilience and mental health. Many of the students experienced traumatic life events and prejudices. One student commented: “And I was born here, so I’m like, I know that I’m American, but I’m not white, so I don’t feel like I’m validated by white people. It’s such a thing with…You have American privilege. I have American privilege for which I’m so grateful, but I don’t have white privilege.”—FG: *peak team*/8th Grade participant (Quote 15, Table 2). Particularly for students who experience privilege and discrimination outside of school, this framework may help mitigate some of the effects of the lived environment for students, providing an alternative exposure. This furthers the principles outlined in positive childhood experiences (PCEs), explained by Dr. Bethell et. al., which are hypothesized to promote a healthy physical and mental adulthood [35]. Our framework expands the framework for community school transformation (CST). The CST initiative is led by the principal and community school coordinator who develop a vision and goal for the school and student [14]. Our framework also highlights not just family engagement, but student-oriented vision and goals. Additionally, our “integrated systems of support” include school-based health as essential efforts for child physical and mental well-being.

The Schools-as-Neighborhoods framework thrives on the concepts of school-based health and social capital, which would address truancy, safety, wellness, and support scholarly exploration. Improving school climate is associated with academic achievement, especially in middle school-age children [36]. Using the School Climate Measure, Daily et al. determined that feeling more connected to their teachers and schools was important for middle school students [36]. High school students’ academic achievement based on these factors was also impacted, although lower than middle school students. Rakesh et al. discuss a restructuring of the child’s brain development with stronger relationships improving mental health [35,37]. These areas require more research to address the differences as students develop, and as priorities and interconnectedness change. Neighborhoods grow with the individual; thus, a School-as-Neighborhood model could support the growth of students throughout their education.

### 4.3. Social Capital Within Schools as Neighborhoods Mitigates Effects of Outside Neighborhood and Promotes Better Learning and Coping Within the School Environment

Social capital is a network of people and resources. An expansive social capital has great impact on an individual’s health, wealth accumulation, and social advancement [38]. Education is a way to expand the social network of students and is a major factor in improving public health outcomes and supporting health equity [32,39]. Our students expressed varying visions for their future life. Most students intend to complete high school and pursue higher education, become entrepreneurs, or just get out of their neighborhoods (Table 2). Their current social capital is highlighted by many having had a loved one die or be incarcerated (Table 1). This negatively impacts the students’ outcomes through social mechanism of contagion and socialization [12]. Contagion is the influence of one’s neighbors on the conduct and beliefs of the individual, while socialization describes the community’s response to those who do not ascribe to the norms of the neighborhood. The school as an ideal neighborhood provides a positive outlook, interrupting the previous socialization.

Teachers and school administrators in our study expressed the ways in which social capital, through the trauma-informed care curriculum and sustained coaching, impacted their students. Students appeared more engaged with a substantial difference in emotional regulation and student demeanor after sustained implementation of the trauma-informed curricula. They stated:

“I like those community-building things. I feel like that always just helps the culture… because during the day there’s a lot of stressful things going on. Sometimes, things get communicated poorly, and people get frustrated with other people, but then it’s at the end of the day, yeah, we’re just all human beings. Playing a goofy game, acting out pictures of someone bowling is really funny, and I think has helped me build better relationships with teachers. I think the staff really engaged well with the students. Students were excited to see staff…When they saw them even outside of the classroom, they were given high fives and greeting them. They really appreciated the energy that all of the staff from STRYV365 brought into our building.”

Teachers noticed differences when the curriculum was paused during summer or winter breaks (Quote 19, 21, Table 3). This suggests that the increase in social capital and a concrete effort to create the culture of the School-as-Neighborhood framework improved behavior and rationale. This may be maintained when students are continuously exposed to a supportive environment. This further shows, as the Robert Wood Johnson Foundation depicts, that a reframing and new environment can have a significant impact on the student. The socialization of the school neighbors can create positive outcomes relationally, but ultimately health-wise, for the student citizens of the school neighborhood [40]. We hypothesize that consistent, relational, trauma-informed interventions help students regulate emotions, feel safer, and engage more deeply in school life. Teachers’ testimonies confirm that school climate improved when these supports were active. Further research accounting for confounding variables is warranted to elucidate the direct impact of the curricula.

### 4.4. School-Based Health as a Form of Connection and Stopgap to Truancy and Low Health Outcomes

Truancy is increased in underserved communities, often due to physical and mental health ailments. It is well documented how the lived environment affects one’s health, with those in underserved areas more likely to have diagnoses of asthma, obesity, anxiety, and depression [41,42]. This is further exacerbated by a student’s inability to attend scheduled doctor’s appointments due to lack of transportation and time constraints [43]. School-based health centers (SBHC) have proven to alleviate these concerns [41]. The Robert Wood Johnson Foundation, a pioneer in public health, first discussed SBH in 1979, piloting nurses in schools to support the success and well-being of children [40]. Lim et al. showed how a visit to a school-based health center increased school attendance, particularly for those who had a mental-health-based visit [2]. Some SBHCs have expanded access to include not only students, but also their families.

SBHCs follow the sentiments of a neighborhood, taking care of the needs of the students and their families, and increasing connectedness [44]. This has proven to increase test scores and graduation rates. However, SBHCs are not present in every state, and in 2023, only 19 states and the District of Columbia had state-allocated funds for SBHCs [44,45]. The ESSA expanded funds to schools based on SBHC as they meet the health and safety goals [3]. Although school-based health is an opportunity to address risk factors for low health outcomes and school performance, Dr. Woolf expresses that healthcare alone does not fully encompass the necessary elements to reduce health inequities [46]. Continuing to identify and create policy impacting the root causes of health inequities ultimately allows for greater success [47]. A reframing of schools as neighborhoods favors a comprehensive view of adolescent education and well-being, building on aspects that have already been proven to support adolescent development, competitiveness, and future goals.

### 4.5. Policy Implications/Recommendations

Initial federal laws and policies focused on expanded access to quality education [1]. The most recent federal education law, signed in 2015—Every Student Succeeds Act (ESSA)—seeks to empower and give autonomy back to states and local educators on improvement metrics and processes [1,3,5]. Although 20% of the distributed federal funds received by districts are to be used for the safety, health of, and support for versatile and adaptable students [5], the implemented funds should be distributed to flexible, locally tailored approaches such as STRYV365 to promote mental health and equity.

State and local policies establish curriculum standards, funding allocation, and daily operations. The addition of social workers and mental health counselors is a state and local practice, which fills support gaps but can sometimes seem disjointed from the school system. There have been various models of schools creating credos and principles that support the values of the whole student. These include charter schools, such as the Knowledge is Power Program (KIPP), and private institutions, Montessori Schools [48,49]. Charter and private schools are given flexibility in classroom methods, which yield positive growth in student reading and math scores, often outperforming traditional public schools. These schools also have on average more days of reading and math learning [31,50]. Although charter schools enroll nearly four million students, this is a fraction of the nearly 55 million students who are registered in K-12 education in the United States [50,51,52].

In Milwaukee, where our four schools, a mixture of public, charter, and private, are located, a discussion about closing Milwaukee public schools causes contention [53,54]. Due to declining rates of enrollment, many school buildings are unoccupied, with others requiring much renovation and expansion. The approved budget for 2025–2026 has Milwaukee Public Schools receiving $1.5 billion [55]. Per student, MPS spends an average of $23,000 for each student, typically larger than other comparable cities. The return on these funds does not appear to be fully realized. As this is a multifaceted, multisystemic issue, a new approach may provide new results. Local and State governments can prioritize revitalizing schools instead of closure. The Schools-as-Neighborhoods framework can support the endeavor. Funding social–emotional and trauma-informed learning programs, such as STRYV365’s curriculum, can create social capital, belonging, and relational safety, making schools a place students want to attend; thus, combating truancy and declining enrollment. Further, school-based health can function as measures of student success and well-being.

### 4.6. Limitations

This study assessed the implementation of a trauma-informed curriculum model that incorporated coaches, peer support, and gamification. The present manuscript intentionally focused on formative and process-oriented findings rather than on definitive estimates of intervention effectiveness. Accordingly, the quantitative results presented in Table 1 are descriptive and intended to characterize the level of adversity and emotional distress in the student population, rather than to test pre-specified impact outcomes. Thus, the study is limited by less robust quantitative evidence. However, anecdotal evidence from focus groups and interviews showed increased interest in school attendance and activities. Future research is needed to further understand the impact of trauma-informed and comprehensive education on truancy and graduation rates. This study utilizes self-reported survey data, which could be attributed to self-selection bias and social desirability bias. Response rates varied across semesters, and missing data were primarily due to absenteeism and student mobility, which may further affect representativeness. The study can be extrapolated through the principles and concepts of a supportive environment where students spend a significant portion of the day. However, the study has restricted generalizability due to its implementation in a mid-sized Midwestern city with a fixed sample size. Our study focuses on middle and high school education; however, students may require various modalities earlier and throughout their education. Future work should quantify behavioral and academic outcomes, exploring the sustainability of this Schools-as-Neighborhoods framework across diverse educational settings. Despite these limitations, this manuscript contributes to the understanding of how trauma-informed, social–emotional curricula promote well-being, especially in at-risk students.

## 5. Conclusions

Schools can function as micro-neighborhoods that nurture belonging, emotional growth, and resilience—key ingredients for educational and mental health success. By reframing schools as neighborhoods, educators and policymakers can create equitable, holistic environments where children thrive physically, academically, emotionally, and socially. Funding should be reallocated to consider and support trauma-informed and positive experiences curricula.

## Figures and Tables

**Table 1:** Survey Results about Adversity by Semester.

Survey Item Responses	Early Fall 2022	Late Fall 2022	Late Spring 2023	Early Fall 2023	Late Fall 2023	Late Spring 2024
Survey participant number	225	277	259	254	223	210
Did you ever have anyone close to you die—yes	74%	73%	72%	73%	74%	69%
Did you every have anyone close to you go to jail—yes	56%	53%	54%	52%	51%	50%
Did you ever have anyone close to you drink or use drugs so often that it caused problems—yes	29%	26%	29%	30%	29%	29%
Over the last 2 weeks, nearly every day and more than half the days have been feeling down, depressed, or hopeless	22%	24%	22%	15%	16%	16%
Over the last 2 weeks, nearly every day and more than half the days have been feeling nervous, anxious, or on edge	30%	24%	23%	20%	18%	20%
Over the last 2 weeks, nearly every day and more than half the days have been bullied, called names, harassed, or abused through texting, social media, or gaming	Not assessed	NA	NA	10%	4%	9%

**Table 2:** Quotes from the students after interacting with the STRYV365 curriculum.

***Overall Comments***
**Quote 1.** “I mean, I like STRYV. It’s just something I look forward to in my day.”—9th Grade, *peak team* **Quote 2.** “Yes, because I actually want to stay longer. So about STRYV? Yeah. Oh, in my opinion, I think STRYV is a really good thing… And STRYV have a lot of things, a lot of activities to do to get your body moving. If you tired, when you go to gym in the morning, you can do the games that they have printed for you and then you would just be, feel like a brand new person.”—FG: *peak team* and Brain Agents/5th grade participant
***Qualities most proud of:***
a. Intrinsic: **Quote 3.** “creativity” “focusing on other people” “being myself” “helpful” “I’m smart” “good person” **Quote 4.** “nice to people” “being on top of my stuff” “keep pushing forward even when it gets hard” **Quote 5.** “controlling my anger” b. Extrinsic: **Quote 6.** “reading” “sports” “athleticism” “energetic” “I can run fast” “I read the Bible” “my hands” **Quote 7.** “artistic” “how clean I am” “I am strong” c. Unable to name quality (negative self-image): **Quote 8.** “I don’t have any.”—8th grade **Quote 9.** “No. Is there a hate myself part?”—FG: *peak team* and Brain Agents/5th grade participant
***Future goal in 5 years (all class years):***
a. Educational Goal **Quote 10.** “college” “learning about cosmetics” “get a degree” “studying as a nurse” “med school” “outside of state” “get a good scholarship” “good grades” “culinary school” “Ivy league” “graduate” “art school” b. Career Goal **Quote 11.** “Orthodontist” “architecture” “doctor” “NBA” “OB” “working at McDonald’s” “summer job” “professional NFL player” “manager at a US Bank” “start my own business” c. Stability Goal **Quote 12.** “getting a car” d. Extracurriculars/Hobbies Goal **Quote 13.** “football” “win the NCAA title” “wrestle” “playing basketball” ‘I’m going to go D1” “work for the Boys and Girls Club” “baseball” e. Other goal **Quote 14.** “stable lifestyle” “apartment” “out of parent’s house” “first car” “get my house” “swimming in money” “Live in Chicago” “driver’s license”
***Neighborhood Cohesion***
**Quote 15.** “And I was born here, so I’m like, I know that I’m American, but I’m not white, so I don’t feel like I’m validated by white people. It’s such a thing with… You have American privilege. I have American privilege for which I’m so grateful, but I don’t have white privilege.”—FG: *peak team*/8th Grade participant

**Table 3:** Quotes from teachers discussing their view of student interactions with the STRYV365 curriculum.

***Teacher Quotes on School/Programming***
***Highlighted Theme:* ***Connection/Community with Schools-Targeted coaching and mentoring* **Quote 16.** “Consistency of Engagement. *peak team* at MAS for 4 or 5 years now (compounding years) I can just say they appreciate when y’all are here, when they’re working with you, they enjoy it… I know the kids are always exciting when they’ve got STRYV for a special…, but… to see the consistency throughout the years, the kids are like, “Oh yeah, you’re that coach, you’re that tall one,” and then, the tall one, that’s always, I think. Good for them.” **Quote 17.** “I think this year too, STRYV Specials came in at a perfect time because I had been out for so long for a period and so for me to come back in and when I didn’t have that bond with my students anymore, the STRYV, especially in the classroom, helped rebuild [00:26:00] that. It really forces them to work as a team…. What a perfect example of why we should have a year-round for everybody, right?” **Quote 18.** “I like those community building things. I feel like that always just helps the culture… because during the day there’s a lot of stressful things going on. Sometimes, things get communicated poorly and people get frustrated with other people, but then it’s at the end of the day, yeah, we’re just all human beings. Playing a goofy game, acting out pictures of someone bowling is really funny, and I think helps, has helped me build better relationships with teachers I think…the staff really engaged well with the students. Students were excited, um, to see staff. They, when they saw them even outside of the classroom, they were given high fives and greeting them. …, they really appreciated the energy that, um, all of the staff from STRYV365 brought into our building” **Quote 19.** “I don’t know that I would have anything to add, um, to that. It was a rough start to the year…second semester was significantly better than first semester. So STRYV may have played some, some part of that, but I don’t, I don’t know that I would have data to defend that one way or the other.” **Quote 20.** “I mean, I think I wish we could have more time than just one special cycle or just one little bit of it. I wish it could be consistent like a regular class. I get it, but that would I think really be powerful too. I think a couple of you guys talked about de-escalation and physical and for me, my vision will be to have them in SEL and then you’re getting those multiple intelligences, the physical, just looking at the screen or just talking because some kids just need to talk to somebody in the morning. I think giving them the choices too on how they start their day… Some kids, they come right in and doing some of those activities with STRYV would give them something positive as well as, like you said, do some de-escalation so they don’t have as much built up by the time they do get to some of those situations.” **Quote 21.** “I think that having more exposure with for them as well would allow them to be able to deescalate. There are peak times of the year where we notice, especially in high school and probably across the board, you get these more infractions of these spikes. People start getting messy, the holidays, breaks, and even during the early parts of the year, even probably being during some summer PD, I don’t know what y’all would go about that but having you all be a little bit more present when those peak seasons come up because we’ve had a brawl at high school.”

## Data Availability

The raw data supporting the conclusions of this article will be made available by the authors on request.

## Abbreviations

The following abbreviations are used in this manuscript:

DoE	Department of Education
SEM	Social Ecological Model
ERIC	Education Resources Information Center
SEL	Social Emotional Learning
EL	Emotional Learning
COPE	Creativity, Optimism, Planning, Expert Information
CRAFFT	Care, Relax, Alone, Forget, Family/Friends, Trouble
NSLP	National School Lunch Program
PCEs	Positive Childhood Experience
SBH	School-based Health
SBHC	School-based health centers
ESSA	Every Student Succeeds Act
KIPP	Knowledge is Power Program

## Appendix A

**Table A1:** Literature Review search terms in Pubmed, ERIC, SCOPUS, and Google Scholar for the Schools-as-Neighborhoods conceptual framework.

Pubmed	“School based health centers” “Social capital” AND “adolescent health”; “social capital” AND “school based health” public school education and research”
ERIC	(((DE “Social Capital” OR DE “Social Environment” OR (TI “social environment” OR “social support”) OR (AB “social environment” OR “social support”)) AND ((TI school*) OR (AB school*) OR DE “Schools” OR DE “Bilingual Schools” OR DE “Boarding Schools” OR DE “Colleges” OR DE “Community Schools” OR DE “Consolidated Schools” OR DE “Correspondence Schools” OR DE “Day Schools” OR DE “Disadvantaged Schools” OR DE “Elementary Schools” OR DE “Experimental Schools” OR DE “Folk Schools” OR DE “Free Schools” OR DE “International Schools” OR DE “Laboratory Schools” OR DE “Magnet Schools” OR DE “Middle Schools” OR DE “Military Schools” OR DE “Montessori Schools” OR DE “Multiunit Schools” OR DE “Neighborhood Schools” OR DE “Open Plan Schools” OR DE “Preschools” OR DE “Private Schools” OR DE “Professional Development Schools” OR DE “Public Schools” OR DE “Racially Balanced Schools” OR DE “Regional Schools” OR DE “Rural Schools” OR DE “Schools of Education” OR DE “Secondary Schools” OR DE “Single Sex Schools” OR DE “Slum Schools” OR DE “Small Schools” OR DE “Special Schools” OR DE “State Schools” OR DE “Suburban Schools” OR DE “Summer Schools” OR DE “Traditional Schools” OR DE “Urban Schools” OR DE “Virtual Schools” OR DE “Vocational Schools” OR DE “Year Round Schools”) OR AB (school* n2 neighborhood*) OR TI (school* n2 neighborhood*)) AND(DE “At Risk Students” OR (AB “at risk student*”) OR (TI “at risk student*”))) AND (DE “School Policy” OR DE “Policy Analysis” OR DE “Economics” OR (TI policy OR policies OR econ* OR financ* OR cost*) OR (AB policy OR policies OR econ* OR financ* OR cost*))
SCOPUS	“urban schools” AND “truancy”; “urban schools” AND “health outcomes”
Google Scholar	“No Child Left Behind” “History of US Education Reform”

## Appendix B

**Table A2:** Incomplete Block Factorial Design for Academic Years 1 and 2 (Time 1 = early fall, Time 2 = after fall semester; Time 3 = after spring semester; BA = Brain Agents; PT = *peak team*; B = both BA and PT; C = control group; X = no participation.

Academic			School			
Year	Grade	Time	A	B	C	D
2022–2023	5	1	C	C	C	X
		2	BA	C	B	X
		3	C	PT	C	X
	6	1	C	C	C	X
		2	C	BA	PT	X
		3	PT	C	C	X
	7	1	C	C	C	X
		2	B	C	C	X
		3	C	BA	PT	X
	8	1	C	C	C	X
		2	C	PT	C	X
		3	BA	C	B	X
	9	1	C	C	C	C
		2	PT	C	BA	C
		3	C	B	C	BA
2023–2024	6	4	C	C	C	X
		5	C	BA	C	X
		6	PT	C	B	X
	7	4	C	C	C	X
		5	BA	C	C	X
		6	C	PT	BA	X
	8	4	C	C	C	X
		5	C	PT	BA	X
		6	B	C	C	X
	9	4	C	C	C	X
		5	PT	C	BA	X
		6	C	BA	PT	X
	10	4	C	C	C	C
		5	C	B	PT	PT
		6	BA	C	BA	C

## Appendix C

**Table A3:** Baseline descriptive statistics of four junior high and high schools.

	School A	School B	School C	School D
**Number of students/scholars**	616	562	365	83
**Race/ethnicity**	51% black, 13% Asian, 9% Hispanic, 17% white, 8% mixed	98% black	54% white, 30% Hispanic, 6% black, 3% Asian, 5% mixed	98% black
**Economic disadvantage**	54%	95%	55%	73%
**English/Language Arts**	36% below basic, 39% basic, 22% proficient	70% below basic, 26% basic	36% below basic, 38% basic, 22% proficient	94% below basic
**Math**	50% below basic, 16% proficient	78% below basic, 18% basic	46% below basic, 32% basic, 20% proficient	97% below basic
**Chronic absenteeism**	21%	42%	20%	17%
**Graduation rate**	95%	86%	90%	47%

**Table A4:** Survey Participant Demographics.

**RACE/ETHNICITY** (Checked all that apply)	*n* = 321
Black or African American	59%
White	35%
Hispanic or Latino	14%
Asian	7%
American Indian or Alaskan Native	5%
Native Hawaiian or Pacific Islander	2%
**GENDER IDENTITY**	*n* = 321
Boy/man	53%
Girl/woman	41%
Non-binary/gender fluid/gender queer/intersex	3%
Prefer not to answer	2%
Other	1%
**CURRENT GRADE LEVEL IN SCHOOL**	*n* = 329
5	14%
6	22%
7	22%
8	16%
9	25%
